# Superoxide-Mediated Upregulation of MMP9 Participates in BMPR2 Destabilization and Pulmonary Hypertension Development

**DOI:** 10.3390/antiox12111961

**Published:** 2023-11-02

**Authors:** Norah Alruwaili, Sharath Kandhi, Ghezal Froogh, Melissa R. Kelly, Dong Sun, Michael S. Wolin

**Affiliations:** 1 Department of Physiology, New York Medical College, Valhalla, NY 10595, USA; norah.ruwaili@gmail.com (N.A.); dong_sun@nymc.edu (D.S.); 2 Department of Basic Sciences, College of Science and Health Professions, King Saud Bin Abdulaziz for Health Sciences, Riyadh 11481, Saudi Arabia

**Keywords:** MMP9, BMPR2, pulmonary hypertension, superoxide, oxidative stress, extracellular matrix, right ventricular hypertrophy

## Abstract

Background and Aims: we previously reported in studies on organoid-cultured bovine pulmonary arteries that pulmonary hypertension (PH) conditions of exposure to hypoxia or endothelin-1 caused a loss of a cartilage oligomeric matrix protein (COMP) stabilization of bone morphogenetic protein receptor-2 (BMPR2) function, a known key process contributing to pulmonary hypertension development. Based on subsequent findings, these conditions were associated with an extracellular superoxide-mediated increase in matrix metalloproteinase 9 (MMP-9) expression. We investigated if this contributed to PH development using mice deficient in MMP9. Results: wild-type (WT) mice exposed to Sugen/Hypoxia (SuHx) to induce PH had increased levels of MMP9 in their lungs. Hemodynamic measures from MMP9 knockout mice (MMP9 KO) indicated they had attenuated PH parameters compared to WT mice based on an ECHO assessment of pulmonary artery pressure, right ventricular systolic pressure, and Fulton index hypertrophy measurements. In vitro vascular reactivity studies showed impaired endothelium-dependent and endothelium-independent NO-associated vasodilatory responses in the pulmonary arteries of SuHx mice and decreased lung levels of COMP and BMPR2 expression. These changes were attenuated in MMP9 KO mice potentially through preserving COMP-dependent stabilization of BMPR2. Innovation: this study supports a new function of superoxide in increasing MMP9 and the associated impairment of BMPR2 in promoting PH development which could be a target for future therapies. Conclusion: superoxide, through promoting increases in MMP9, mediates BMPR2 depletion and its consequent control of vascular function in response to PH mediators and the SuHx mouse model of PH.

## 1. Introduction

Remodeling of the vascular extracellular matrix is a significant pathological feature of pulmonary hypertension (PH), a progressive disease characterized by elevated blood pressure in the pulmonary circulation (≥20 mmHg) leading to dysfunction of the right ventricle [1]. We previously reported that in organoid-cultured bovine pulmonary arteries (BPA), pulmonary hypertension (PH) mediators hypoxia or endothelin-1 induced an increase in superoxide levels and deficiency of cartilage oligomeric matrix protein (COMP) affecting the bone morphogenetic protein receptor-2 (BMPR2) function [2,3]. Superoxide elevation is implicated in vascular remodeling by activating several pathophysiological mechanisms such as cellular migration, proliferation, increased extracellular matrix turnover, and recruitment of inflammatory cells [4]. Recently, it has been reported that in vitro exposure to hypoxia significantly increased pulmonary artery vascular remodeling which was associated with increased levels of matrix metalloproteinas-9 (MMP9) [5]. MMP9 is suggested to play a pivotal role in cardiovascular diseases due to its ability to disrupt the basement membrane of endothelial cells and the degradation of several types of matrix proteins [6]. Superoxide has been shown to activate MMP9 potentially by dissociating the cysteine–zinc interaction between the pro-peptide and the active site of MMP9, allowing the release of the active enzyme [7]. Thus, we hypothesized that superoxide-mediated MMP9 activation facilitates the progression of PH. Hence, investigating its role yields a promising area for developing new approaches to reverse vascular remodeling and enhancing responsiveness to pulmonary vasodilators in established PH.

## 2. Materials and Methods

All salts used for making physiological solutions were analyzed reagent grade from Baker Chemical. All gases were purchased from Airgas (Bronx, NY, USA). Sugen5416 was obtained from Adooq Bioscience LLC (Irvine, CA, USA, Catalog No. A12437), Endothelin-1 was obtained from Sigma-Aldrich (Burlington, MA, USA. Catalog No. E7764), and FK-506 was purchased from Cayman Chemical (Ann Arbor, MI, USA, Catalog No. 10007965). Specific antibodies were purchased from the companies indicated: anti-MMP9 (Abcam, Cambridge, MA, USA: ab38898), anti-BMPR2 (Abclonal, Woburn, MA, USA: A16778), anti-COMP (Abclonal, Woburn, MA, USA: A13963) anti-GC (Sigma, Burlington, MA, USA: G4405), anti-SIRT3 (Abcam, Cambridge, MA: ab86671), anti-SOD2 (Abclonal, Woburn, MA, USA: A1340) and anti-β-actin (Sigma, Burlington, MA, USA: A5441). Superoxide scavengers and detection probes were purchased from the companies indicated: bovine SOD (Sigma, Burlington, MA, USA: S7571), Tempo (Sigma, Burlington, MA, USA; 214000).

Animal studies: all experiments were performed following the New York Medical College Animal Care and Use Committee-approved protocol, in accordance with the National Institutes of Health Guidelines for the Care and Use of Laboratory Animals. Adult male and female (10–12-week-old) Matrix Metalloproteinase 9 (MMP9) knockout mice and appropriate age-matched wild-type (WT) controls were used. All mice were purchased from Jackson Laboratories (Bar Harbor, ME, USA).

Chronic hypoxia exposure protocol: mice were either exposed to normoxic (21% O_2_) or normobaric (10% O_2_) hypoxic conditions for 21 days in a hypoxic in vivo cabinet (Coy Laboratories, Grass Lake, MI, USA) in NYMC animal facility care employing adaptations of methods previously described [2]. In this study, the combination of sugen and chronic hypoxia model was used to induce pulmonary hypertension. Mice were injected subcutaneously with VEGFR2 inhibitor Sugen5416 suspended in DMSO (20 mg/kg) once a week according to a published protocol [8]. At the end of the experimental protocol, mice were sacrificed with inhalation of 100% carbon dioxide, and then lungs and hearts were harvested from each mouse to be processed further for studies. In some experimental groups, mice were injected with DMSO or Sugen under normoxic conditions to serve as negative control mice.

### 2.1. Right Heart Catheterization

Right heart catheterization was used to assess right ventricular systolic pressure (RVSP) in wild-type and MMP9 KO mice using adaptations of methods previously described [9]. After echocardiography analysis, the anesthetized mouse was transferred to a heating plate to maintain the body temperature at 37 °C. A middle incision was made on the neck to expose the right external jugular vein. A 1.2 F solid-state catheter (Transonic Scisense Inc., London, ON, Canada) was inserted into the jugular vein and advanced into RV to monitor RVSP. Observing a stable ventricular pressure wave was indicative of the accurate position of the catheter in the RV. RVSP was recorded using a PowerLab data acquisition system (ADInstruments, Colorado Springs, CO, USA) and analyzed with LabChart V8 software (ADInstruments).

### 2.2. Doppler Echocardiographic Measurements

Transthoracic echocardiography was performed on the mice that were under light anesthesia through a constant flow of isoflurane, employing adaptations of methods previously described [9]. A mechanical transducer set at 30 MHz (Vevo 770; Visualsonics, Toronto, ON, Canada) was used to obtain an aortic B-mode image of the heart. The pulsed-wave Doppler sampler was positioned on the pulmonary valve leaflets and aligned with the direction of the flow for obtaining a pulsed-wave Doppler recording of the pulmonary blood flow. The pulmonary blood flow parameters measured included: (1) pulmonary artery acceleration time (PAAT), the time from the onset of the pulmonary flow to peak velocity using pulsed-wave Doppler recordings; (2) ejection time (ET), the time from the onset to the end of the systolic flow; (3) the PAAT to ET ratio, an index of pulmonary arterial pressure that corresponds inversely with the severity of pulmonary hypertension; and (4) velocity–time integral (VTI), the integral of the area under the captured image of Doppler flow of the pulmonary valve’s velocity–time wave was calculated using a mechanical transducer set at 30 MHz (Vevo 770; Visualsonics) for each experimental group under a constant flow of isoflurane. Heart rate was recorded with electrocardiograph electrodes located on the platform, and it was adjusted with isoflurane in order to keep it constant throughout the procedure.

### 2.3. Measurement of Reactivity in Mouse Pulmonary Arteries

Freshly prepared pulmonary arteries were used for studies measuring changes in isometric force, conducted initially in an atmosphere of 21% O_2_ and 5% CO_2_ in Krebs-bicarbonate buffer at 37 °C, employing adaptations of methods previously described [10]. Pulmonary arterial rings were mounted on Danish Myograph Technology wire myographs, with a Powerlab data acquisition system from ADInstruments used to record time-dependent changes in force. Pulmonary rings were incubated at optimized passive tensions of 0.5 g for one hour in Krebs-bicarbonate buffer containing 118 mM NaCl, 4.7 mM KCl, 1.5 mM CaCl_2_, 25 mM NaHCO_3_, 1.1 mM MgSO_4_, 1.2 mM KH_2_PO_4_, and 5.6 mM glucose gassed with 21% O_2_-5% CO_2_-74% N_2_ to maintain a pH of 7.4. Following this one hour of incubation, the rings were depolarized with 123 mM KCl containing Krebs-bicarbonate buffer, and the rings were again re-equilibrated with standard Krebs-bicarbonate buffer for another 30 min before being contracted with 10^−7^ M Phenylephrine (PE). The normal endothelial function of vessel rings was evaluated with a dose-dependent relaxation to Acetylcholine (ACh; 10^−8^ M–10^−5^ M) under a phenylephrine-induced pre-contraction, while other rings were treated with a dose-dependent relation to spermine-NONOate (10^−9^ to 10^−5^ M) to evaluate endothelium-independent vasodilation. After re-equilibration of the vessels in Krebs solution for an additional 30 min, vessel rings that received ACh were incubated with 1 μM SOD, and the ones treated with NONOate received 0.5 μM TEMPO for 30 min before repeating the same experimental protocols.

Tissue preparation: bovine lungs were obtained from a slaughterhouse in ice-cold phosphate buffer saline. The second or third main branches of bovine pulmonary arteries (BPAs) were used in the experiments, employing adaptations of methods previously described [11]. Bovine arteries were cleaned of their connective tissue and then cut into rings of 2–3 mm in diameter and width. The endothelium was removed by rubbing the lumen. As indicated in the results, bovine rings were organoid cultured in the absence and presence of different agents including 100 nM ET-1, 1 μM SOD, and FK506 (0.1 μM), with Dulbecco’s modified Eagle medium containing 10% fetal bovine serum and 1% antibiotics (penicillin, streptomycin, and amphotericin B) for 24 or 48 h at 37 °C with 5% CO_2_.

Western blot analysis: frozen lung tissues or endothelium-removed bovine pulmonary arteries were pulverized and then homogenized in lysis buffer containing protease and phosphatase inhibitors, using adaptations of methods previously described [3]. The Bradford method was used to assay protein quantification, and samples were prepared for gel electrophoresis. Proteins were separated using a 10% SDS-polyacrylamide gel under reducing and denaturing conditions. Gels were transferred to polyvinylidene difluoride membranes, and the membranes were blocked with Tris-buffered saline with Tween 20 + 5% milk for one hour. The membranes were then incubated with primary and secondary antibodies as per the manufacturer’s protocol. Protein bands were visualized with an enhanced chemiluminescence kit (Pierce, Rockford, IL, USA) on X-OMAT autoradiography paper (Kodak, Rochester, NY, USA) in a dark room. Protein levels were measured using densitometry analysis with the UN-SCAN-IT version 7.1 gel software by Silk Scientific (Orem, UT, USA).

Superoxide measurements using chemiluminescence and cytochrome C reduction methods: changes in superoxide were measured from quantifying the chemiluminescence of 5 μM lucigenin in a liquid scintillation counter (LS6000IC; Beckman Instruments, San Diego, CA, USA) with a single active photomultiplier tube in a dark room, using adaptations of methods previously described [9]. Initial background chemiluminescence (blank readings) was measured in plastic scintillation mini vials containing only 5 μM lucigenin in 1 mL of Krebs solution buffered with 10 mM HEPES-NaOH (pH 7.4) in the absence of tissue. Right after obtaining the blank readings, arterial rings were added into each vial to measure the chemiluminescence in the presence of the tissue (tissue readings). The blank measurement was subtracted from subsequent measurements taken in the presence of arterial rings to give an indication of the total superoxide levels. To obtain an estimate of the extracellular superoxide levels in the rings, 1 μM SOD was added to the vials, and the subsequent reading was recorded. Tissue measurement was subtracted from the SOD measurement of each ring, and the difference was considered an estimation of the extracellular superoxide levels. The rings were weighed at the end of the experiment. The counts were divided by weight to gain the final data in counts per minute per mg of tissue. The reduction of cytochrome C (75 μM, in the presence of 1 μM catalase) with its increase in absorbance at 550 nm over 3 h in the absence and presence of 1 μM SOD was also used to quantify extracellular superoxide release from organoid-cultured BPA rings.

Superoxide measurement using HPLC: measurement of the superoxide-specific hydroxylated products of Mitosox and dihydroethidium (DHE) is previously described [2] for quantifying changes in mitochondrial and extra-mitochondrial superoxide using an HPLC system with a Jasco FP-1520 fluorescence detector and a Beckman ultrasphere reverse column (C18; 5 μm, 250 × 4.6 mm). The fluorescence excitation and emission wavelengths used for Mitosox detection are 510 and 595, and for DHE, the wavelengths are 480 and 580.

Statistical Analysis: statistical analysis was performed using Graph Pad Prism 5 software. All values are expressed as means ±  SEM, with n and data points shown in figures equal to the number of animals studied. Statistical analyses between the two groups were conducted with paired or unpaired Student’s *t*-test; one-way ANOVA or two-way ANOVA were used for comparison between multiple groups, as described in the results. *p* < 0.05 was used to establish statistical significance.

## 3. Results

### 3.1. MMP9 Contributes to the Pathogenesis of Sugen/Hypoxia-Induced Pulmonary Hypertension

**Figure 1:**
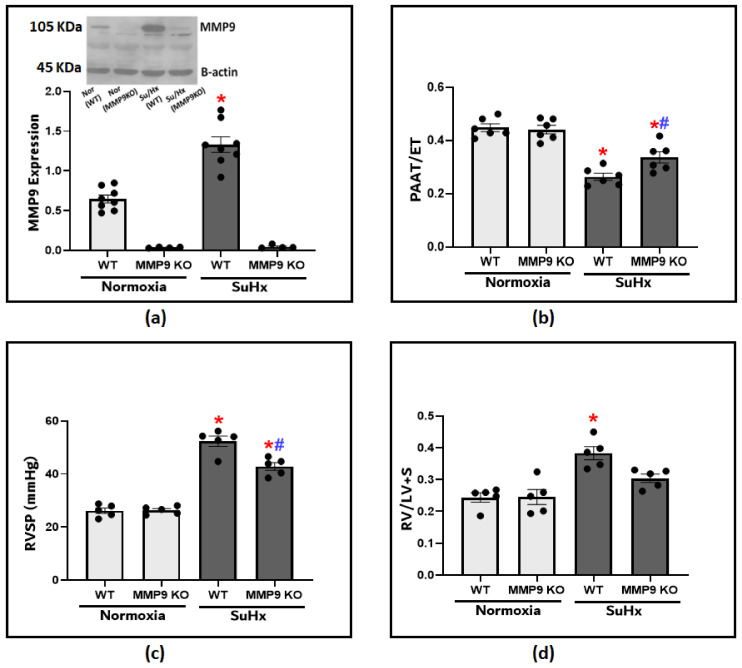
(**a**) Showing the representative blot and quantitative measurement of MMP9 expression in lung tissues of wild-type and MMP9 knockout mice exposed to normoxia or Sugen plus chronic hypoxia for 3 weeks. (**b**) The effect of Sugen/hypoxia on right ventricular systolic pressure (RVSP) via right heart catheterization in wild-type and MMP9 KO mice. (**c**) Echocardiographic measurements of pulmonary artery acceleration time (PAAT) to ejection time (ET) ratio in wild-type and MMP9 knockout mice as an indication of pulmonary arterial pressure at the end of the experimental protocol. (**d**) Right ventricular hypertrophy in wild-type and MMP9 knockout mice as indicated by the ratio of the weight of the right ventricle (RV) to the left ventricle plus the septum (LV + S). (*n* = 4–8) * *p* < 0.05 vs. Normoxia (WT); # *p* < 0.05 vs. WT (SuHx) using one-way ANOVA with Tukey’s multiple comparison test.

We utilized an established experimental protocol composed of the administration of Sugen5416 plus hypoxia exposure (SuHx) to recapitulate the development of PH. After 3 weeks, MMP9 protein levels in the lung tissues were significantly increased compared to those of the normoxic WT control mice (Figure 1a). To investigate MMP9 contribution in the pathogenesis of PH, we used MM9 KO mice to assess if inducing MMP9 deficiency shows any protective effects on the development of the disease model. Doppler echocardiography provided a non-invasive method to assess pulmonary hemodynamic under conditions of minimized heart rate changes among the groups by adjusting the delivery of isoflurane. A lower PAAT is an indication of higher pulmonary artery pressures (PAP), and the ratio of PAAT/ejection time (ET) was used as a reliable estimate for PH development (Figure 1b). The echocardiographic assessment showed a significant decrease in pulmonary artery acceleration time (PAAT) in the SuHx-WT mice compared to the SuHx-MMP9 KO group or the control mice. After that, we administered right heart catheterization, which is the gold standard method for diagnosing pulmonary hypertension. Our findings document that there was a significant increase in right ventricular systolic pressure (RVSP) in response to SuHx exposure, indicating that increased PAP is potentially caused by elevated pulmonary vascular resistance (Figure 1c). RVSP in SuHx-MMP9 KO mice was substantially lower compared to their wild-type SuHx group. Moreover, RVSP elevation was accompanied by RV hypertrophy, as documented with an observed increase in the Fulton index, calculated from the percent ratios of the right ventricular/left ventricular plus septum weights (Figure 1d). Thus, the data in Figure 1 document that PH parameters in SuHx-MMP9 KO mice were attenuated when compared to those of SuHx-WT mice. These findings suggest that MMP9 has a prominent role in altering the pulmonary hemodynamics observed in this SuHx mouse model of PH.

### 3.2. Vascular Regulation Alterations of Pulmonary Arteries from Mice with SuHx-Induced PH Are Partly Dependent of MMP9 Expression

#### 3.2.1. Impairment of Endothelium-Dependent Vasodilation

**Figure 2:**
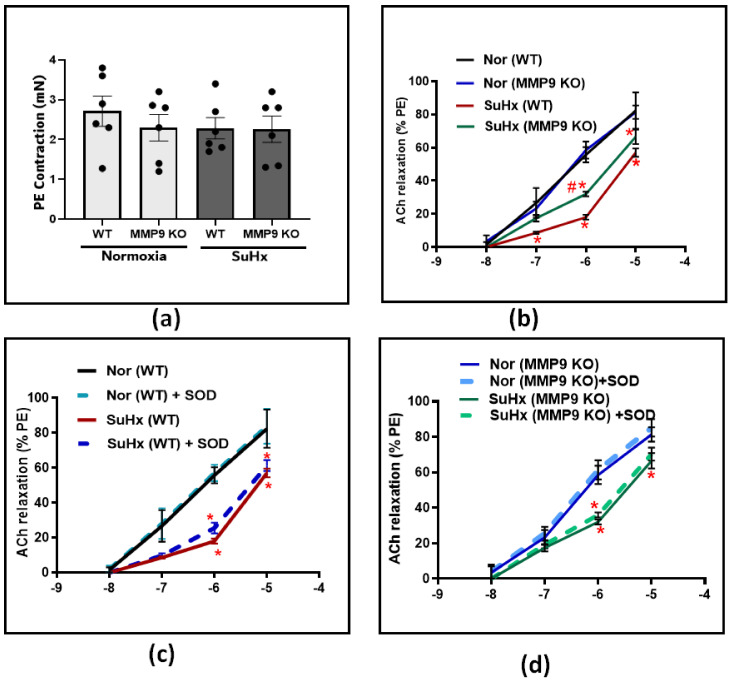
(**a**) Summary data showing the contractile force induced by Phenylephrine (PE) at 10^−7^ M in pulmonary arteries from all groups. (**b**) Summary data for acetylcholine-dose-dependent relaxation in pulmonary arteries from wild-type and MMP9 KO mice exposed to normoxia and Sugen plus chronic hypoxia. (**c**) The effect of acute treatment of SOD (1 μM) on the acetylcholine-relaxation response in pulmonary arteries from wild-type mice exposed to normoxia or Sugen plus chronic hypoxia. (**d**) Summary data showing the effect of acute treatment of SOD (1 μM) on Ach relaxation response in pulmonary arteries from MMP9 KO mice exposed to normoxia or Sugen plus chronic hypoxia. (*n* = 4) * *p* < 0.05 vs. corresponding normoxic group; using two-way ANOVA with Tukey’s multiple comparison test. # *p* < 0.05 vs. WT (SuHx) mice; using unpaired *t*-test.

Following the hemodynamic assessment, vascular studies were conducted to investigate the effect of MMP9 deficiency on pulmonary artery tone regulation. Pulmonary arteries from all experimental groups were dissected into equal-sized circular rings and precontracted with 10^−7^ M PE. After that, the rings were treated with increasing doses of an acetylcholine (ACh) in the presence and absence of the superoxide scavenger SOD. In PE-precontracted pulmonary artery rings, our results show that the contraction force induced by PE was similar among all the groups (Figure 2a). In response to increasing cumulative doses of ACh, the concentration-dependent relaxation did not differ between WT and MMP9 KO mice exposed chronically to the normoxia control conditions examined for subsequent comparison with exposure to conditions promoting PH. In contrast, the ACh response was significantly less in the SuHx WT group compared to that of the mice exposed to normoxic conditions. However, in SuHx MMP9 KO mice, there was significant protection against the loss of endothelium-dependent vasodilation, specifically at ACh doses of 10^−7^ and 10^−6^ (Figure 2b). These findings suggest either decreased production of, or sensitivity to, the endothelium-derived vasodilators, such as NO in the setting of our experimental model. For a better understanding of the restorative mechanism induced by MMP9 depletion against endothelial dysfunction, we incubated the pulmonary arteries with SOD prior to ACh-induced relaxation. The results shown in Figure 2c,d demonstrated that scavenging superoxide using SOD treatment had minimal detectable effects on the concentration-dependent relaxation of pulmonary arteries to ACh in all of the experimental groups of mice.

#### 3.2.2. Impairment of Endothelium-Independent Vasodilation

**Figure 3:**
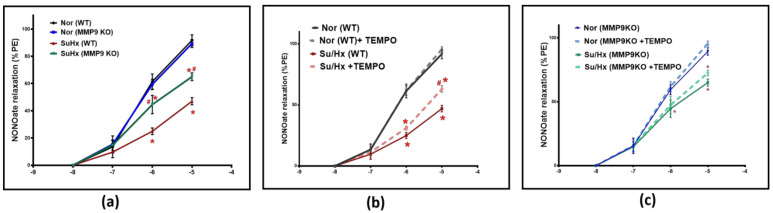
(**a**) Summary data for NONOate dose-dependent relaxation in pulmonary arteries of wild-type and MMP9 KO mice exposed to normoxia and Sugen plus chronic hypoxia. (**b**) Summary data showing the effect of acute treatment of TEMPO (0.5 μM) on NONOate relaxation responses in pulmonary arteries of wild-type mice exposed to normoxia or Sugen plus chronic hypoxia. (**c**) Summary data showing the effect of acute treatment of TEMPO (0.5 μM) on the NONOate relaxation response in pulmonary arteries of MMP9 KO mice exposed to normoxia or Sugen plus chronic hypoxia. (*n* = 5) * *p* < 0.05 vs. corresponding Normoxic group; using two-way ANOVA with Tukey’s multiple comparison test. # *p* < 0.05 vs. Su/Hx group using one-way ANOVA with Tukey’s multiple comparison test.

To further illustrate the role of MMP9 on vascular tone, pulmonary arteries were treated with increasing concentrations of a NO donor relaxing agent, spermine NONOate; (10^−8^–10^−5^ M). NONOate treatment showed a significantly less vasodilatory response in the SuHx WT mice compared to their control group. Conversely, the SuHx MMP9 KO mice showed a significantly larger NONOate relaxation response compared to the SuHx-treated WT mice, suggesting an increased responsiveness of the VSMCs to NO-mediated vasodilators in arteries from SuHx PH mice deficient in MMP9 (Figure 3a). In separate experiments, pulmonary arteries were relaxed with NONOate in the presence and absence of the superoxide scavenger 0.5 µM TEMPO to examine if superoxide interaction with NO is a potential mechanism for the observed decrease in relaxation. Our findings showed that TEMPO treatment led to an elevation in NONOate-mediated vasodilatory response in SuHx WT mice that is significant only at the 10^−5^ M dose compared to blood vessels examined without TEMPO treatment (Figure 3b). Interestingly, TEMPO treatment did not alter the loss of NONOate relaxation seen in the MMP9 KO mice under the same experimental protocol. Thus, our findings from the vascular studies (Figure 3b vs. Figure 3c) showed that the direct effects on the vasodilatory response by adding TEMPO in these doses of NONOate only support detecting a prevention by MMP9 depletion of the minor inhibitory effect of acute scavenging of NO by superoxide at a NONOate dose of 10^−5^ M. In contrast, the SuHx MMP9 KO did not show a similar response to TEMPO, suggesting that MMP9 regulates the source of superoxide that has acute effects on impairing NO-mediated relaxation. These findings provide evidence for an additional mechanism in the pulmonary arteries of SuHx mice impairing relaxation to NO which appears to be independent of an acute scavenging of NO by superoxide.

### 3.3. Upregulation of MMP9 Expression Promoted BMPR2 Depletion in PH

**Figure 4:**
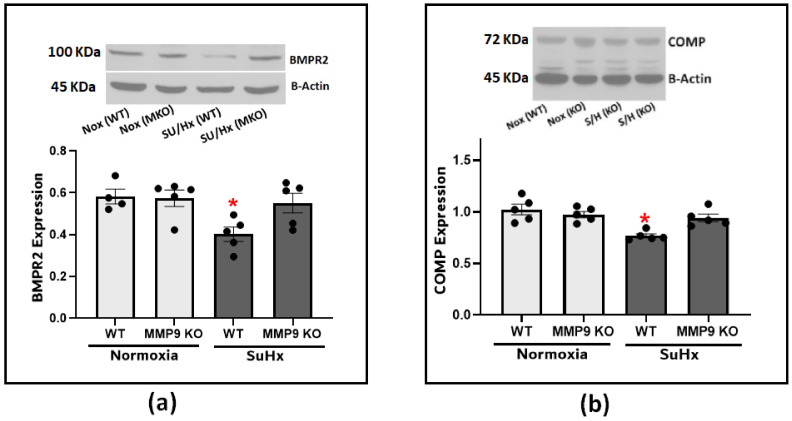
(**a**) Western blot analysis for BMPR2 expression in lung tissues from wild-type and MMP9 KO mice exposed to normoxia or Sugen plus hypoxia. (**b**) Western blot analysis for COMP expression in lung tissues from wild-type and MMP9 KO mice exposed to normoxia or Sugen plus hypoxia. (*n =* 4–7) * *p* < 0.05 vs. Nor (WT) using one-way ANOVA with Tukey’s multiple comparison test.

Next, we looked into the effect of MMP9 on BMPR2 expression considering its established role in protecting the integrity of the pulmonary blood vessels. Our results confirmed the effect of BMPR2 on the development of PH as there was a significant depletion of BMPR2 in the lung tissues from the SuHx-induced PH compared to the control mice (Figure 4a). The observed depletion was significantly attenuated in the SuHx MMP9 KO mice, indicating that MMP9 activation plays a role in BMPR2 depletion. Since little is known about the interplay between MMP9 and BMPR2, and our prior studies have noted the importance of COMP in regulating vascular functions through its stabilizing interaction with BMPR2, we examined COMP levels to investigate its potential to be cleaved by MMP9 and to confirm our previous findings linking COMP depletion to alteration of superoxide levels [2]. The results of our experiments found clear support that an MMP9 deficiency seems to protect against COMP depletion induced by SuHx PH (Figure 4b).

### 3.4. PH Mediators, Endothelin-1 and Hypoxia, Induce MMP9 Expression via a Superoxide-Mediated Pathway

**Figure 5:**
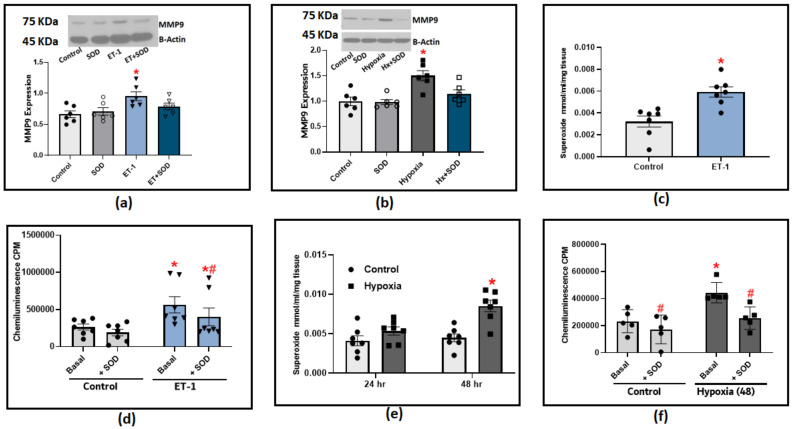
The effect of incubating bovine pulmonary arteries with ET-1 (100 nM) or under hypoxic conditions (3%O_2_ balance N_2_). (**a**) MMP9 expression in BPA incubated with ET-1 for 24 h in the presence or absence of 1 μM SOD. (**b**) MMP9 expression in BPA incubated under hypoxic conditions for 48 h in the presence or absence of 1 μM SOD. (**c**) Superoxide production response to ET-1 treatment in BPA as measured with cytochrome c reduction assay. (**d**) Superoxide detection with 5 μM lucigenin in BPA incubated with ET-1 for 24 h before and after adding 1 μM SOD. (**e**) Superoxide detection by the SOD inhibition of cytochrome c reduction assay in BPA incubated in hypoxia for 24 and 48 h (*n* = 7). (**f**) Superoxide detection with 5 μM lucigenin in BPA incubated under hypoxic conditions (3%O_2_) for 48 h before and after adding 1 μM SOD. (*n* = 5–7) * *p* < 0.05 vs. Control; # *p* < 0.05 vs. corresponding group with the same treatment using one-way ANOVA with Tukey’s multiple comparison test.

The following experiments were designed to determine the effect of known PH mediators, hypoxia and Endothelin-1 (ET-1), on MMP9 expression. Incubating organoid-cultured BPA with ET-1 induced a significant increase in MMP9 expression (Figure 5a). In BPA incubated under hypoxic conditions (3%O_2_ balance N_2_), we only observed a significant increase in MMP9 levels after 48 h (Figure 5b). A strong relationship between MMP9 elevation and superoxide generation has been reported in the literature. Therefore, we utilized two redox measurement methods to specifically detect changes in superoxide levels in response to hypoxia and ET-1. The organoid culture of BPA treated with ET-1 (100 nM) for 24 h significantly increased the extracellular superoxide levels as detected with the measurement of the SOD-inhibited reduction of cytochrome C measured by the increase in its absorbance at 550 nm (Figure 5c). Moreover, we applied the lucigenin chemiluminescence assay to further confirm the effect of ET-1. Our findings with lucigenin shown in Figure 5d were in parallel with the cytochrome C reduction assay data in which ET-1 treatment appeared to double the extracellular superoxide levels detected using both methods compared to control BPA. In this experiment, adding exogenous SOD (1 μM) is essential to allow us to differentiate the contribution of extracellular superoxide levels detected by lucigenin chemiluminescence compared to superoxide detected from intracellular sources. The chemiluminescence data shows that around 20% of the ET-1-induced chemiluminescence increase detects superoxide generation directed toward the extracellular matrix. Incubating BPA under hypoxia conditions elicited a similar response in which superoxide production was significantly increased in cytochrome C reduction assay (Figure 5e) and lucigenin chemiluminescence assay (Figure 5e). To further dissect the role of superoxide on MMP9 induction in response to PH mediators, we added exogenous SOD (1 μM) to the previously described organoid culture combinations of ET-1 or hypoxia. We then examined the effects of scavenging extracellular superoxide on MMP9 expression. The results shown in Figure 5a,b indicate that scavenging extracellular superoxide with added SOD significantly reduced the increase in MMP9 levels, supporting that extracellular superoxide is a contributing factor in the increased MMP9 associated with ET-1 and hypoxia treatments in BPA. Collectively, our results show that hypoxia and ET-1 can trigger different signaling mechanisms that could potentially regulate MMP9 on transcriptional as well as post-translational levels, which are both known to show a ROS-dependent activation.

### 3.5. BMPR2 Stabilization Protected against Endothelin-1-Induced Dysfunction in Pulmonary Arteries

**Figure 6:**
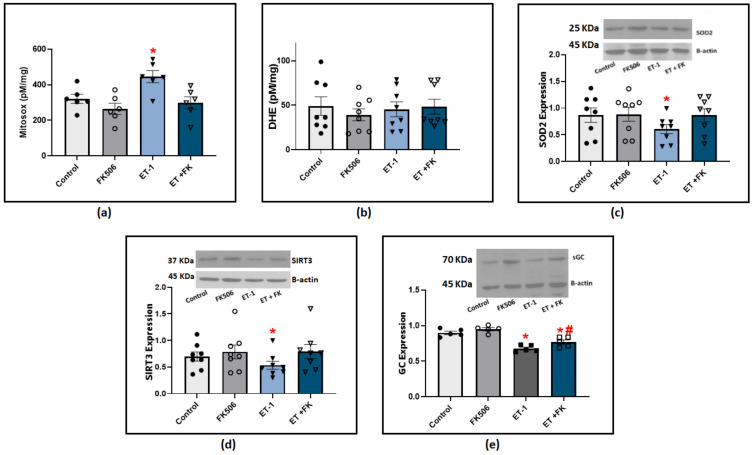
(**a**) Summary data for mitochondrial matrix superoxide levels detected using Mitosox in BPA organoid cultured for 24 h with (100 nM) ET-1 in the presence or absence of (0.1 μM) FK506. (**b**) Summary data for extra-mitochondrial matrix superoxide levels detected using DHE in BPA incubated with ET-1 in the presence or absence of FK506. (**c**) Western blot analysis showing SOD2 expression in BPA organoid cultured for 24 h with ET-1 in the presence and absence of FK506. (**d**) Western blot analysis of SIRT3 expression in BPA organoid cultured with ET-1 in the presence and absence of FK506. (**e**) Western blot analysis of sGC expression in BPA organoid cultured for 24 h with ET-1 in the presence and absence of (0.1 uM) FK506. (*n* = 5–8) * *p* < 0.05 vs. control; # *p* < 0.05 vs. ET-1 using two-way ANOVA with Tukey’s multiple comparisons test.

Our previous studies showed that both ET-1 and hypoxia stimulate the production of ROS, primarily superoxide anion (O_2_^−^), and this is associated with a loss of COMP stabilization of BMPR2 regulation of oxidant-linked pathophysiological processes potentially related to PH development [2,3]. We further investigated if BMPR2 stabilization by FK506 influenced ET-1 modulation of superoxide levels. Our results from BPA cultured with ET-1 showed a significant elevation of superoxide levels indicated by HPLC quantification of Mitosox oxidation products (Figure 6a). These data support the detection of an increase in superoxide levels in the mitochondrial matrix. We also measured DHE oxidation products under similar experimental conditions, and we did not detect a significant difference among the groups treated with ET-1 or FK506 (Figure 6b). These results suggested that incubating BPA with ET-1 in the presence of FK506 may prevent ET-1-induced impairment of other aspects of mitochondrial function in our preparation. In order to define the mechanism of ET-1-induced elevation in mitochondrial superoxide levels, we examined the expression of SOD2, the main superoxide scavenger in the mitochondrial matrix. Our results indicated a significant decrease in SOD2 levels in response to ET-1 incubation in BPA, and the treatment of FK506 protected against this loss of SOD2 (Figure 6c). Sirtuin 3 (SIRT3) has been found to regulate mitochondrial antioxidant response and function. We looked into its expression to examine if ET-1 potentially influences mitochondrial superoxide and SOD2 via modulating SIRT3 levels. Our results indicate that incubation of endothelium-removed BPA with ET-1 significantly reduced SIRT3 levels, which was restored to control levels in BPA treated with a combination of ET-1 and FK506 (Figure 6d). We also examined if rescuing BMPR2 restored endothelin-induced impairment of soluble guanylate cyclase (sGC). NO beneficial effects are mediated by the activation of its intracellular receptor sGC and subsequent cGMP signaling pathways. Western blot analysis indicated that ET-1 incubation caused a significant decrease in sGC expression, which was significantly suppressed in the presence of FK506 (Figure 6e). Collectively, our results suggest that FK506 protects against ET-1-induced elevation of mitochondrial superoxide and promotes a protective regulatory effect on the sGC/NO pathway via BMPR2-mediated stabilization. While scatter was observed in the data for individual conditions studied across animals in the ex vivo studies from bovine tissues, the two-way ANOVA analysis used compensated for this variability, resulting in statistics documenting the consistent pattern changes observed in the responses of arterial segments from the same animal.

## 4. Discussion

In this study, the loss of MMP9 in mice was observed to significantly attenuate the development of SuHx-induced PH, suggesting a pivotal role for MMP9 in the pathological process of this disease model. While the mechanisms of MMP9 function are not entirely understood, it has been suggested to potentially involve proteolytic release and the activation of factors, with much of our understanding of its biological activities originating from knockout studies. For example, MMP9 has been shown to be a key player in inducing angiogenesis, based on observations of the MMP9 KO mice showing an abnormal pattern of skeletal growth plate vascularization and ossification [12]. In the present study of a PH disease model, our findings are consistent with reports showing that treating mice with SU5416 in combination with chronic hypoxia led to a severe form of PH, characterized by a significant elevation of RVSP and PA pressure compared to the normoxic control mice. Vascular dysfunction characterized by impaired vasodilatory mechanisms and increased pulmonary arteriolar medial wall thickening can be contributing factors to the development of these observations. One interesting finding is that the SuHx disease model showed a significant increase in MMP9 expression. MMP9 elevation can be explained in part by increased levels of inflammatory cytokines, specifically interleukin (IL)-6 and tumor necrosis factor-α (TNFα), both of which have been reported to induce MMP9 levels in different disease models. Inflammatory markers seem to participate significantly in the pathogenesis mechanism of pulmonary hypertension [13]. An alternative explanation for these results could be due to an immune response, which could function upstream and/or downstream to phenotypically alter vascular endothelial and vascular smooth muscle cells [14,15]. By comparing SuHx groups, RVSP was elevated in both WT and MMP9 KO mice. However, it was significantly higher in SuHx WT compared to SuHx MMP9 KO mice. A similar pattern of results was obtained for the PAAT/ET ratio in these two groups, with SuHx mice having lower values than SuHx MMP9 KO mice, indicating lower PA pressure in the absence of MMP9. This difference suggests that MMP9 deficiency seems to have beneficial effects which decrease the progression of PH. The observed right ventricular hypertrophy in SuHx mice represents an adaptive response to improve the ventricle’s ability to deal with the progressive elevation of afterload. MMP9 KO mice treated with SuHx showed evidence of decreased pulmonary artery pressure compared to the control mice, and this consequently decreased the afterload response and attenuated the remodeling of right ventricular tissues compared to their SuHx WT littermates. Another finding that stands out is the absence of cardiac dysfunction in MMP9 KO under normoxic conditions, suggesting that MMP9 physiological levels are kept at a minimum and are only induced by pathological triggers.

Many aspects of PH pathogenesis could be attributed to an imbalance between vasoconstrictors and vasodilators in pulmonary arteries. MMP9 appears to promote disturbances in pulmonary vascular tone regulation. In vascular studies where acetylcholine was used, Sugen hypoxia treatment significantly reduced the relaxation response, indicating the involvement of endothelial dysfunction in the development of the disease, which was partially mitigated by MMP9 deletion (Figure 2). The importance of the endothelium in regulating vascular tone was first established when relaxation induced by acetylcholine was only observed when the endothelial lining was intact. Since then, the endothelium has been shown to regulate vascular tone, either partially or entirely, by releasing NO in response to acetylcholine [16], and many other processes regulate the release of NO from endothelium. The partial effect of MMP9 deficiency in restoring ACh relaxation responses is consistent with the partial protection of MMP9 deficiency in the SuHx mouse model of responses to NONOate and the in vivo protection against vascular pathogenesis seen in this model. The impairment of ACh-induced relaxation in SuHx could involve many factors other than MMP9 regulation, even though other reports suggested a more prominent role of MMP9 in endothelial dysfunction [17]. It should be noted that these reports were mainly investigating MMP9 levels in endothelial cells from systemic blood vessels, which can explain any differences with our findings.

The relaxation responses to NONOate shown in Figure 3 were also significantly decreased in the SuHx group compared with those in the control group. However, the loss of this relaxation response was attenuated in the MMP9 KO group. These findings suggest that the deletion of MMP9 may be beneficial in protecting against PH in vivo because a deficiency in MMP9 prevents depletion or dysfunction of proteins, contributing to key aspects of vascular function such as the nitric oxide–guanylate cyclase pathway [18]. In vascular smooth muscle cells, NO activates soluble guanylyl cyclase (sGC) and increases cGMP to levels that increase cGMP-dependent protein kinase (PKG) activity, which is kinase that regulates multiple mechanisms contributing to vasodilation [19]. Peroxynitrite, the product of NO and superoxide anion, can cause a loss of the Fe^2+^-heme receptor site on soluble guanylate cyclase (sGC) for NO, either by promoting sGC heme oxidation (potentially causing a depletion of sGC), or by decreasing the availability of heme through mitochondrial matrix superoxide, inhibiting heme biosynthesis by ferrochelatase [3].

While the acute treatment of pulmonary arteries isolated from SuHx mice with SOD did not significantly improve relaxation to ACh, treatment with TEMPO promoted a statistically significant, small partial restoration in the endothelium-independent vasodilatory response to the 10^−5^ M dose of NONOate. This indicates a detectable potential role for the removal of superoxide in regulating vascular relaxation by NO perhaps through an acute attenuation of superoxide, resulting in increased bioavailability of NO in an intracellular region not accessible to extracellular SOD. Consistent with these observations is evidence by Sheak et al., reporting that TEMPOL reduced U-46619-induced vasoconstriction compared to control levels in neonatal chronic hypoxia-induced pulmonary hypertension [20]. Overall, our results demonstrate only a modest effect of acute superoxide scavenging of NO, influencing its bioavailability. Although the impairment of vascular tone regulation has been the foundation for many of the current treatments of PH by either enhancing vasodilation or suppressing vasoconstriction pathways [21,22], alteration of vascular tone is not the only pathogenic feature of vascular dysfunction in PH. Hence, it is also possible that the hemodynamic improvements induced by the MMP9 loss in our study may be caused by decreased inflammatory cell infiltration and edema, leading to a possible suppression of the pathologic remodeling process, independent of its vasodilatory effect. Wang et al. reported that MMP9 promoted a pro-inflammatory autocrine loop in VSMCs through the regulation of cytokine expression mediated by fibrinogen [23], while another study showed that MMP9 siRNA was successful in impairing a TNFα/NF-κB signaling pathway in endothelial cells [24]. Our present study goes beyond previous reports by showing that MMP9 contributes to the loss of the protective effect of COMP on BMPR2. Factors modulating the expression of BMPR2 are of particular importance because it is the most dominant genetic mutation that is linked directly to patients of pulmonary hypertension [25]. In this study, MMP9 KO mice treated with Sugen plus chromic hypoxia showed almost complete prevention of the decreased BMPR2 levels observed in the wild-type mice exposed to the same experimental protocol. These observations indicate that MMP9 activation contributes to the loss of BMPR2 observed in this animal model of PH, either by direct interaction or indirectly by cleaving proteins essential for stabilization and/or activation of BMPR2. We have previously reported that COMP provides a protective effect against hypoxia-induced BMPR2 depletion [2]. COMP is an essential molecule that functions to maintain healthy vascular blood vessels. It plays a critical role in maintaining the contractile phenotype of pulmonary arterial smooth muscle associated with suppression of hypoxia-induced increases in superoxide production. Also, lung tissues from rodents exposed to chronic hypoxia show reduced levels of COMP.

In the SuHx animal model, which exhibits a more severe form of pulmonary hypertension, there is an activation of MMP9-mediated cleavage of COMP and subsequently loss of its stabilization of BMPR2. This compromised effect is possibly due to superoxide-related inflammatory responses further promoting an induction of IL-6 and TNFα. Thus, our study provides a possible mechanism for COMP depletion in vivo where COMP serves as a substrate of MMP9 based on the findings from MMP9-deficient mice showing a preserved expression of COMP when exposed to SuHx (Figure 4). Other matrix proteinases could act to destabilize BMPR2 via a similar mechanism. A study by Hurst et al. reported that ADAM10 and ADAM17 participated in BMPR2 cleavage in pulmonary artery smooth muscle cells (PASMCs) which was mediated by TNFα [26]. Although they did not investigate MMP9 activity in the previous study, other reports confirmed that TNFα is a potent activator of MMP9 expression in different types of tissues [27].

MMP9 regulation is a complex process, and our in vitro study in endothelium-rubbed BPA (Figure 5) documented a possible contribution of extracellular superoxide in modulating MMP9 levels in response to the PH mediators hypoxia and endothelin-1. Superoxide can react with NO to produce peroxynitrite. The latter has been reported to control MMP9 activity through S-nitrosylation of cysteine thiols [28], and to disrupt the Zn-thiolate bond in pro-MMP-9 leading to its activation [29]. Moreover, our data showed that pulmonary arteries treated with ET-1 are presented with increased levels of mitochondrial matrix superoxide (Figure 6). This elevation can result from epigenetic or posttranslational suppression of the mitochondrial superoxide dismutase (SOD2). A study conducted by Archer et al. reported that SOD2 methylation is a potential mechanism for the development of PH. Their data showed that SOD2 knockdown by siRNA was sufficient to create a PAH phenotype in normal PASMCs [30]. SIRT3 post-translationally maintains SOD2 activity via deacetylation, significantly enhancing its ability to scavenge ROS [31]. Our findings showed that endotelin-1 significantly decreased the expression of SIRT3 and SOD2 (Figure 6) in pulmonary arteries. These detrimental effects seem to be mediated by the impairment of BMPR2 signaling pathways based on the results showing that FK506, a BMPR2 stabilizer, successfully reversed these effects.

The activation of guanylate cyclase (GC) by NO leads to the formation of the second messenger cGMP, which mediates various physiological processes in pulmonary arteries and protective responses against vascular injury, promoting remodeling. NO activates sGC by binding to the ferrous heme cofactor; hence, oxidative stress is a major factor influencing enzyme activity. The observed significant elevation of mitochondrial superoxide (Figure 6a) acutely reduces NO bioactivity through a loss of the Fe^2+^-heme receptor site on soluble guanylate cyclase (sGC) for NO, either by promoting sGC heme oxidation (potentially causing a depletion of sGC), or by decreasing the availability of heme through mitochondrial matrix superoxide, inhibiting heme biosynthesis [3]. Vascular studies with acute treatment with TEMPO (Figure 3) showed that eliminating superoxide only modestly restored the NO vasodilatory response. Thus, oxidation of the sGC heme and/or the observed depletion of sGC may be major factors contributing to the loss of relaxation and beneficial effects of NO-sGC-cGMP regulation in the models of PH examined in this study.

**Figure 7:**
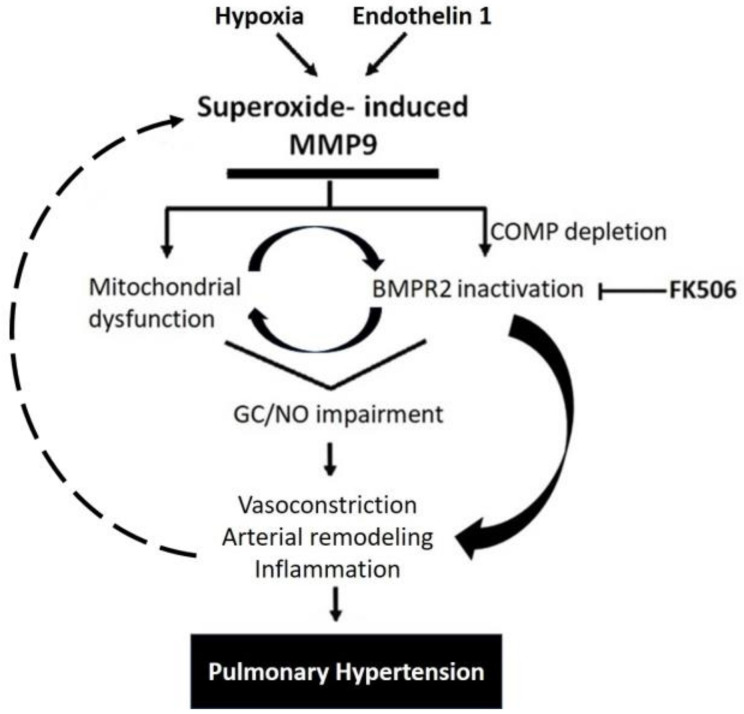
Schematic representation showing MMP9 proposed mechanisms involved in pulmonary hypertension.

Interestingly, some studies showed that inhibiting MMP9 reversed the production of ROS, indicating that MMP9 can act upstream of ROS generation [32,33]. In the same manner, a decrease in MMP9 activity can cause a reduction in oxidative stress, probably via a feedforward mechanism [34]. Altogether, the findings of our study support the induction of MMP9 levels in response to specific PH mediators that contribute to many pathological aspects observed in pulmonary arteries, as summerized in the proposed model shown in Figure 7. The model illustrates how a depletion of BMPE2 via the elevation of MMP9 can contribute to a feedforward elevation of superoxide and pathophysiological processes contributing to PH progression.

## 5. Conclusions

Collectively, our results cast a light on new aspects of how MMP9 functions in pulmonary blood vessels and provide evidence for its critical role in vascular dysfunction. We documented roles for superoxide, triggering a pathological feed-forward loop of MMP9 induction associated with the loss of COMP and/or BMPR2 in pulmonary arteries, caused by the incubation of PH mediators or in an animal model of PH. Disturbance of this pathological loop at any level provides a tractable approach for therapeutic intervention. Although our findings demonstrated that MMP9 deletion yields significantly better outcomes in regard to PH development, a few limitations should be noted for our present study. Firstly, it is possible that MMP9 knockout may alter immune and inflammatory pathways as part of its physiological response. Hence, further study is warranted to examine these effects in MMP9 knockout mice. Secondly, future translational studies on humans are needed to support a clinical application.

## Data Availability

All data are presented in the manuscript. The data presented in this study are available on request from the corresponding author.

## References

[1] Humbert M., Kovacs G., Hoeper M.M., Badagliacca R., Berger R.M., Brida M., Carlsen J., Coats A.J., Escribano-Subias P., Ferrari P. (2022). 2022 ESC/ERS Guidelines for the diagnosis and treatment of pulmonary hypertension: Developed by the task force for the diagnosis and treatment of pulmonary hypertension of the European Society of Cardiology (ESC) and the European Respiratory Society (ERS). Endorsed by the International Society for Heart and Lung Transplantation (ISHLT) and the European Reference Network on rare respiratory diseases (ERN-LUNG). Eur. Heart J..

[2] Yu H., Alruwaili N., Hu B., Kelly M.R., Zhang B., Sun D., Wolin M.S. (2019). Potential role of cartilage oligomeric matrix protein in the modulation of pulmonary arterial smooth muscle superoxide by hypoxia. Am. J. Physiol. Lung Cell Mol. Physiol..

[3] Yu H., Alruwaili N., Kelly M.R., Zhang B., Liu A., Wang Y., Sun D., Wolin M.S. (2022). Endothelin-1 depletion of cartilage oligomeric matrix protein modulates pulmonary artery superoxide and iron metabolism-associated mitochondrial heme biosynthesis. Am. J. Physiol. Lung Cell Mol. Physiol..

[4] Tabima D.M., Frizzell S., Gladwin M.T. (2012). Reactive oxygen and nitrogen species in pulmonary hypertension. Free Radic. Biol. Med..

[5] Cao X., He Y., Li X., Xu Y., Liu X. (2019). The IRE1α-XBP1 pathway function in hypoxia-induced pulmonary vascular remodeling, is upregulated by quercetin, inhibits apoptosis and partially reverses the effect of quercetin in PASMCs. Am. J. Transl. Res..

[6] Yabluchanskiy A., Ma Y., Iyer R.P., Hall M.E., Lindsey M.L. (2013). Matrix metalloproteinase-9: Many shades of function in cardiovascular disease. Physiology.

[7] Rajagopalan S., Meng X.P., Ramasamy S., Harrison D.G., Galis Z.S. (1996). Reactive oxygen species produced by macrophage-derived foam cells regulate the activity of vascular matrix metalloproteinases In Vitro. Implications for atherosclerotic plaque stability. J. Clin. Invest..

[8] Vitali S.H., Hansmann G., Rose C., Fernandez-Gonzalez A., Scheid A., Mitsialis S.A., Kourembanas S. (2014). The Sugen 5416/hypoxia mouse model of pulmonary hypertension revisited: Long-term follow-up. Pulm. Circ..

[9] Kandhi S., Alruwaili N., Wolin M.S., Sun D., Huang A. (2019). Reciprocal actions of constrictor prostanoids and superoxide in chronic hypoxia-induced pulmonary hypertension: Roles of EETs. Pulm. Circ..

[10] Patel D., Alhawaj R., Kelly M.R., Accarino J.J., Lakhkar A., Gupte S.A., Sun D., Wolin M.S. (2016). Potential role of mitochondrial superoxide decreasing ferrochelatase and heme in coronary artery soluble guanylate cyclase depletion by angiotensin II. Am. J. Physiol. Heart Circ. Physiol..

[11] Gupte S.A., Wolin M.S. (2006). Hypoxia promotes relaxation of bovine coronary arteries through lowering cytosolic NADPH. Am. J. Physiol. Heart Circ. Physiol..

[12] Vu T.H., Shipley J.M., Bergers G., Berger J.E., Helms J.A., Hanahan D., Shapiro S.D., Senior R.M., Werb Z. (1998). MMP-9/gelatinase B is a key regulator of growth plate angiogenesis and apoptosis of hypertrophic chondrocytes. Cell.

[13] Otsuki S., Sawada H., Yodoya N., Shinohara T., Kato T., Ohashi H., Zhang E., Imanaka-Yoshida K., Shimpo H., Maruyama K. (2015). Potential contribution of phenotypically modulated smooth muscle cells and related inflammation in the development of experimental obstructive pulmonary vasculopathy in rats. PLoS ONE.

[14] Alexander M.R., Owens G.K. (2012). Epigenetic control of smooth muscle cell differentiation and phenotypic switching in vascular development and disease. Annu. Rev. Physiol..

[15] Schermuly R.T., Ghofrani H.A., Wilkins M.R., Grimminger F. (2011). Mechanisms of disease: Pulmonary arterial hypertension. Nat. Rev. Cardiol..

[16] Furchgott R.F., Zawadzki J.V. (1980). The obligatory role of endothelial cells in the relaxation of arterial smooth muscle by acetylcholine. Nature.

[17] Florence J.M., Krupa A., Booshehri L.M., Allen T.C., Kurdowska A.K. (2017). Metalloproteinase-9 contributes to endothelial dysfunction in atherosclerosis via protease activated receptor-1. PLoS ONE.

[18] Mam V., Tanbe A.F., Vitali S.H., Arons E., Christou H.A., Khalil R.A. (2010). Impaired vasoconstriction and nitric oxide-mediated relaxation in pulmonary arteries of hypoxia-and monocrotaline-induced pulmonary hypertensive rats. J. Pharmacol. Exp. Ther..

[19] Walford G., Loscalzo J. (2003). Nitric oxide in vascular biology. J. Thromb. Haemost..

[20] Sheak J.R., Yan S., Weise-Cross L., Ahmadian R., Walker B.R., Jernigan N.L., Resta T.C. (2020). PKCβ and reactive oxygen species mediate enhanced pulmonary vasoconstrictor reactivity following chronic hypoxia in neonatal rats. Am. J. Physiol. Heart Circ. Physiol..

[21] Lee J.E., Hillier S.C., Knoderer C.A. (2008). Use of sildenafil to facilitate weaning from inhaled nitric oxide in children with pulmonary hypertension following surgery for congenital heart disease. J. Intensive Care Med..

[22] Burger C.D. (2009). Pulmonary hypertension in COPD: A review and consideration of the role of arterial vasodilators. COPD J. Chronic Obstr. Pulm. Dis..

[23] Wang S., Liu J., Wu D.I., Pang X., Zhao J., Zhang X. (2015). Pro-inflammatory effect of fibrinogen on vascular smooth muscle cells by regulating the expression of PPARα, PPARγ and MMP-9. Biomed. Rep..

[24] Ramella M., Boccafoschi F., Bellofatto K., Follenzi A., Fusaro L., Boldorini R., Casella F., Porta C., Settembrini P., Cannas M. (2017). Endothelial MMP-9 drives the inflammatory response in abdominal aortic aneurysm (AAA). Am. J. Transl. Res..

[25] Lane K.B., Machado R.D., Pauciulo M.W., Thomson J.R., Phillips J.A., Loyd J.E., Nichols W.C., Trembath R.C. (2000). Heterozygous germline mutations in BMPR2, encoding a TGF-β receptor, cause familial primary pulmonary hypertension. Nat. Genet..

[26] Hurst L.A., Dunmore B.J., Long L., Crosby A., Al-Lamki R., Deighton J., Southwood M., Yang X., Nikolic M.Z., Herrera B. (2017). TNFα drives pulmonary arterial hypertension by suppressing the BMP type-II receptor and altering NOTCH signalling. Nat. Commun..

[27] Ramnath R., Foster R.R., Qiu Y., Cope G., Butler M.J., Salmon A.H., Mathieson P.W., Coward R.J., Welsh G.I., Satchell S.C. (2014). Matrix metalloproteinase 9-mediated shedding of syndecan 4 in response to tumor necrosis factor α: A contributor to endothelial cell glycocalyx dysfunction. FASEB J..

[28] Okamoto T., Akaike T., Sawa T., Miyamoto Y., van der Vliet A., Maeda H. (2001). Activation of Matrix Metalloproteinases by Peroxynitrite-induced Protein S-Glutathiolation via Disulfide S-Oxide Formation. J. Biol. Chem..

[29] St. Croix C.M., Wasserloos K.J., Dineley K.E., Reynolds I.J., Levitan E.S., Pitt B.R. (2002). Nitric oxide-induced changes in intracellular zinc homeostasis are mediated by metallothionein/thionein. Am. J. Physiol. Lung Cell Mol. Physiol..

[30] Archer S.L., Marsboom G., Cipriani N., Rehman J., Kim G.H., Zhang H.J., Toth P.T., Svensson E.C., Dyck J.R.B., Gomberg-Maitland M. (2010). Epigenetic attenuation of mitochondrial superoxide dismutase 2 in pulmonary arterial hypertension: A basis for excessive cell proliferation and a new therapeutic target. Circulation.

[31] Tao R., Coleman M.C., Pennington J.D., Ozden O., Park S., Jiang H., Kim H., Flynn C.R., Hill S., Hayes McDonald W. (2010). Sirt3-Mediated Deacetylation of Evolutionarily Conserved Lysine 122 Regulates MnSOD Activity in Response to Stress. Mol. Cell.

[32] Kalani A., Pushpakumar S.B., Vacek J.C., Tyagi S.C., Tyagi N. (2016). Inhibition of MMP-9 attenuates hypertensive cerebrovascular dysfunction in Dahl salt-sensitive rats. Mol. Cell Biochem..

[33] Touyz R.M. (2006). Mitochondrial Redox Control of Matrix Metalloproteinase Signaling in Resistance Arteries. Arterioscler. Thromb. Vasc. Biol..

[34] Gasche Y., Copin J., Sugawara T., Fujimura M., Chan P.H. (2001). Matrix Metalloproteinase Inhibition Prevents Oxidative Stress-Associated Blood-Brain Barrier Disruption After Transient Focal Cerebral Ischemia. J. Cereb. Blood Flow Metab..

